# The arginine synthetase pathway is responsible for carbamoyl phosphate synthesis in *Thermococcus kodakarensis*

**DOI:** 10.3389/fmicb.2026.1761990

**Published:** 2026-05-29

**Authors:** Yuta Michimori, Naoki Ohashi, Claudia Szymanski, Haruyuki Atomi

**Affiliations:** 1 Department of Synthetic Chemistry and Biological Chemistry, Graduate School of Engineering, Kyoto University, Kyoto, Japan; 2 Department of Chemical Science and Engineering, Graduate School of Engineering, Kyoto University, Kyoto, Japan; 3 Integrated Research Center for Carbon Negative Science, Kyoto University, Kyoto, Japan

**Keywords:** Archaea, arginine, carbamoyl phosphate, pyrimidine, Thermococcales

## Abstract

Carbamoyl phosphate is considered a precursor for arginine and pyrimidine nucleotide biosynthesis in all domains of life. Carbamoyl phosphate is formed from bicarbonate and ammonia by carbamoyl phosphate synthetase (CPS) at the expense of two ATPs. Although CPS homologs are widespread across archaea, many members of Thermococcales, including *Thermococcus kodakarensis*, do not harbor these homologs, leaving the source of carbamoyl phosphate unresolved. Here we show that in *T. kodakarensis*, carbamoyl phosphate is supplied from arginine via the recently identified arginine synthetase pathway. Disruption of *arcE*, encoding arginine synthetase, or *arcB*, encoding ornithine transcarbamoylase, caused severe growth defects in pyrimidine-free medium, whereas disruption of *arcC*, encoding carbamate kinase, had little effect. Under growth conditions dependent on hydrogenase activity, *arcC* disruption again had little effect, whereas growth of the *arcB* disruption strain was not observed. Growth was partially restored by heterologous expression of a CPS homolog from *Pyrococcus chitonophagus*. The same construct also partially restored growth of *arcE* and *arcB* disruption strains under pyrimidine-free conditions. Comparative genomic analysis revealed that while pyrimidine biosynthesis and [NiFe]-hydrogenases are universally conserved in Thermococcales, canonical carbamoyl phosphate synthetase is absent from most members, implying that the arginine synthetase pathway serves as the principal source of carbamoyl phosphate in this archaeal order.

## 1 Introduction

**Figure 1:**
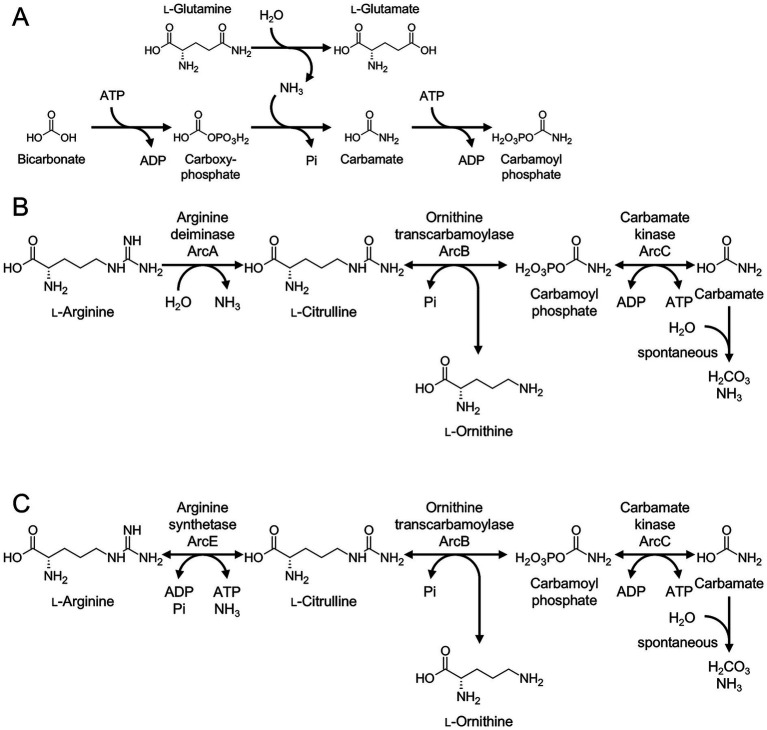
Reaction catalyzed by carbamoyl phosphate synthetase and arginine degradation pathways. **(A)** Carbamoyl phosphate (CP) is synthesized from bicarbonate and ammonia in a two-step, ATP-dependent reaction catalyzed by carbamoyl phosphate synthetase (CPS). Ammonia can be supplied either as free ammonia or released from glutamine by the glutamine-hydrolyzing small subunit of CPS. In the first step, bicarbonate and ammonia are condensed to form carbamate in an ATP-dependent manner. In the second step, carbamate is phosphorylated by a second ATP molecule to produce CP. **(B)** Arginine deiminase pathway. Arginine is first converted to citrulline and ammonia by arginine deiminase (ArcA/ADI). Citrulline is then subjected to a phosphorolysis reaction catalyzed by ornithine transcarbamoylase (ArcB/OTC) to produce ornithine and CP. Carbamate kinase (ArcC/CK) subsequently transfers the phosphate of CP to ADP, yielding ATP and carbamate, which decomposes to ammonia and bicarbonate. **(C)** Arginine synthetase pathway in *T. kodakarensis*. Arginine synthetase (ArcE; TK2200) couples the deimination of arginine directly to ATP synthesis, generating citrulline, ammonia, and ATP from ADP and inorganic phosphate. Citrulline is then cleaved by OTC (ArcB; TK0871) to ornithine and CP. CP is utilized to generate ATP by CK (ArcC; TK2158).

Carbamoyl phosphate (CP) is a precursor for the biosynthesis of biomolecules including arginine and pyrimidines. CP is synthesized by carbamoyl phosphate synthetase (CPS) in a two-step reaction using bicarbonate, ammonia (either free or derived from glutamine), and two molecules of ATP. First, an ATP-dependent condensation of bicarbonate and ammonia forms carbamate, followed by phosphorylation of carbamate using a second ATP (Figure 1A) (Metzenberg et al., 1957; Anderson and Meister, 1965). CP is utilized for the carbamoylation of ornithine by ornithine transcarbamoylase (OTC) for arginine biosynthesis (Supplementary Figure 1A) (Burnett and Cohen, 1957; Charlier and Glansdorff, 2004) and aspartic acid by aspartate transcarbamoylase for pyrimidine biosynthesis (Supplementary Figure 1B) (Lowenstein and Cohen, 1956; Jensen et al., 2008). An *Escherichia coli* strain lacking CPS exhibits auxotrophy for arginine and pyrimidine (Alcántara et al., 2000). In canonical pyrimidine *de novo* biosynthesis, aspartate transcarbamoylase initiates formation of the pyrimidine ring by converting CP and aspartate to carbamoyl aspartate. Carbamoyl aspartate is subsequently converted to uridine 5′-monophosphate (UMP) via reactions catalyzed by dihydroorotase, dihydroorotate dehydrogenase, orotate phosphoribosyltransferase, and orotidine-5′-phosphate decarboxylase, encoded by *pyrF* (Supplementary Figure 1B).

CP is also required to generate the NiFe(CN)₂(CO) active site of [NiFe] hydrogenases, which is composed of Ni and Fe cations along with one CO and two CN^−^ ligands (Ogata et al., 2016; Sawers et al., 2025) (Supplementary Figure 2). In *E. coli*, disruption of CPS genes abolished hydrogenase activity, suggesting that CP is required for the formation of the active site (Paschos et al., 2001). Isotopic labeling and biochemical analyses revealed that the two CN^−^ ligands originate from the carbamoyl group of CP (Reissmann et al., 2003). The crystal structures of the hydrogenase maturation protein HypE in both the carbamoylated and thiocyanated forms suggested a stepwise conversion of CP-derived carbamoyl moieties into CN^−^ ligands (Tominaga et al., 2013).

In terms of catabolism, CP is utilized by carbamate kinase (CK) to generate ATP and conserve energy. CK is a key component of the arginine deiminase (ADI) pathway. The ADI pathway proceeds via the stepwise conversion of arginine and water to citrulline and ammonia by ADI (ArcA), citrulline and phosphate to ornithine and CP by OTC (ArcB), and CP and ADP to carbamate and ATP by CK (ArcC). Carbamate is spontaneously hydrolyzed to ammonia and bicarbonate in aqueous solution (Figure 1B) (Cunin et al., 1986). It can thus be assumed that CK catalyzes the reverse reaction of the second reaction of CPS, the ATP-dependent phosphorylation of carbamate. However, CK and CPS proteins are not structurally related to one another.

The ADI pathway is widely distributed in prokaryotes, and has been studied in organisms including the bacterium *Pseudomonas aeruginosa* (Wauven et al., 1984) and the archaeon *Halobacterium salinarum* (Hartmann et al., 1980; Ruepp and Soppa, 1996). The pathway enables anaerobic growth on arginine through substrate-level phosphorylation by the CK reaction. In the hyperthermophilic archaeon *Thermococcus kodakarensis*, ADI is replaced with arginine synthetase (ArcE), an enzyme that conserves the thermal energy released in the conventional ADI reaction (~37.7 kJ mol^−1^) to generate ATP from ADP and phosphate (Michimori et al., 2024). Thus in this pathway, designated the arginine synthetase pathway (Figure 1C), arginine can be converted to ornithine and CP, as in the ADI pathway. However, the breakdown of a single arginine leads to the generation of two ATPs in the arginine synthetase pathway. ArcE homologs are widely distributed in archaea and bacteria, and among organisms that harbor ArcC, the occurrence of ArcA and ArcE complement one another. The occurrence of ArcA or ArcE is not related to those of argininosuccinate synthetase and argininosuccinate lyase genes, responsible for arginine biosynthesis from citrulline. Instead, we have observed a tendency that ArcE is present in organisms that are known to utilize amino acids and peptides for catabolism. The advantage of ArcE over ArcA in organisms that mainly utilize sugars as the catabolic substrate may be limited, and explain the persistence of ArcA in nature.

In members of Thermococcales, CP generation from bicarbonate and ammonia was detected in cell extracts of *Pyrococcus furiosus* (Legrain et al., 1995) and *Pyrococcus abyssi* (Purcarea et al., 1996), suggesting that these species possess a CPS. However, the protein responsible for CP generation in these species is still under discussion. The CK proteins from *P. furiosus* and *P. abyssi* display primary structures common to previously characterized CKs, and the three-dimensional structure of the CK protein from *P. furiosus* was similar to those of typical CKs (Uriarte et al., 1999; Ramón-Maiques et al., 2000). However, several lines of evidence raised the possibility that the CK protein is responsible for CP synthesis instead of CPS. A CPS homolog is not found on the *P. abyssi* genome, and the recombinant CK from *P. abyssi*, with concentrations of 120 mM NH_4_Cl and 60 mM NaHCO_3_, displayed CP-generating activity (Purcarea et al., 2001). *E. coli* strains lacking their endogenous CPS but expressing *P. furiosus* CK were able to grow in minimal medium lacking arginine and pyrimidines when supplemented with 20–50 mM ammonium bicarbonate (Alcántara et al., 2000). Although CP-generating activity has only been observed at extremely high concentrations of bicarbonate and ammonia, the involvement of CK in CP generation cannot be ruled out, and thus the enzyme responsible for CP biosynthesis in Thermococcales has yet to be firmly established. Intriguingly, members of Thermococcales that harbor CPS homologs are limited. CPS homologs are only found in seven species including *P. furiosus* (PF1713, PF1714) (Robb et al., 2001), *Pyrococcus chitonophagus* (CHITON_1946, CHITON_1945) (Papadimitriou et al., 2016) and *Thermococcus argininiproducens* (K1720_00595, K1720_00600) (Park et al., 2023) (Supplementary Table 1). In contrast, homologs cannot be found on the *P. abyssi* and *T. kodakarensis* genomes, raising questions on how these species supply CP for arginine/pyrimidine metabolism and [NiFe] hydrogenase maturation. The physiological contribution of CK to CP biosynthesis in Thermococcales, and the true nature of the CP supply routes in species without CPS, therefore remain unresolved. To address these issues, genetic analyses of CK and CPS in Thermococcales are required, as previously emphasized (Alcántara et al., 2000).

*T. kodakarensis* is capable of *de novo* synthesis of pyrimidine bases (Sato et al., 2003) and hydrogen production by [NiFe] hydrogenase (Kanai et al., 2011). However, although *T. kodakarensis* possesses a CK homolog (ArcC, TK2158), genes encoding CPS are not present on its genome. This raises the possibility that arginine is the major source for CP generation via ArcE and ArcB in this organism instead of ammonium and bicarbonate. In order to identify the metabolic source of CP in *T. kodakarensis* and gain insight on the roles of CK and CPS proteins in members of Thermococcales, here we evaluated the phenotypes of strains disrupted of or introduced with genes involved in CP metabolism. The results suggest that ArcE and ArcB play key roles in supplying CP from arginine in *T. kodakarensis*. Furthermore, our results suggest that in Thermococcales species that harbor canonical CPS homologs, CPS rather than CK plays a major role in CP generation from ammonium and bicarbonate.

## 2 Results

### 2.1 Candidate genes involved in CP metabolism

We carried out a search for candidate genes that were involved in the production or consumption of CP. As previously reported (Michimori et al., 2024), arginine synthetase (ArcE), OTC (ArcB) and CK (ArcC) are encoded by TK2200, TK0871 and TK2158, respectively (Supplementary Table 1). Genes with homology to arginine deiminase and CPS are not present on the *T. kodakarensis* genome. This gene occurrence is similar to that found in *P. abyssi*, and represents a genotype corresponding to those of *P. furiosus*, *Pyrococcus woesei* and *P. chitonophagus* disrupted of their CPS genes. Using *T. kodakarensis* as a host cell, here we examine the effects of disrupting TK2200, TK0871 or TK2158, and also study the effects of introducing a CPS gene from *P. chitonophagus* into *T. kodakarensis*. Phenotypic effects were evaluated by examining pyrimidine auxotrophy and hydrogenase-dependent growth.

### 2.2 Disruption of *arcE* or *arcB* results in pyrimidine auxotrophy

**Figure 2:**
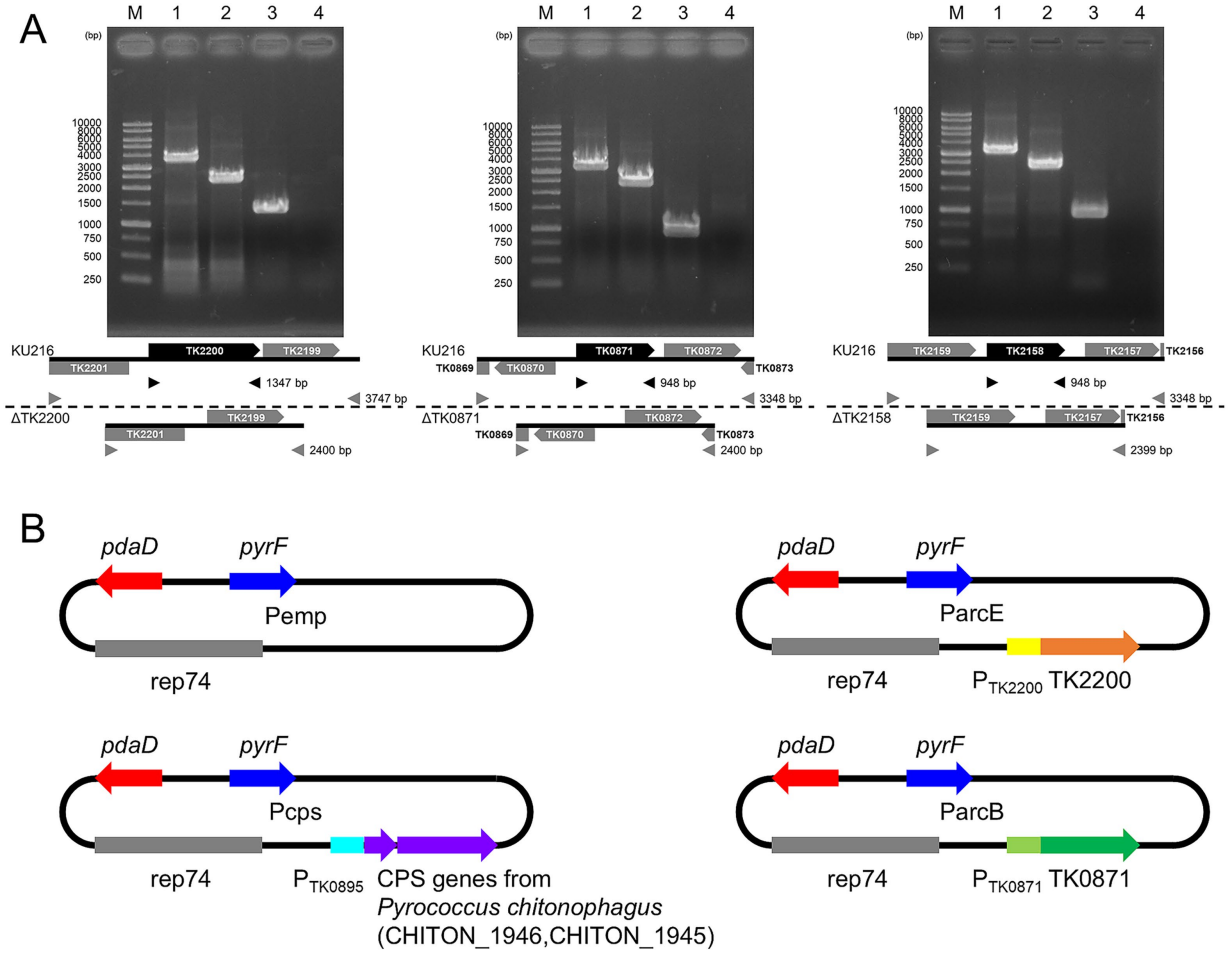
PCR analysis of gene disruption strains and schematic illustrations of plasmids. **(A)** The genotypes of individual gene disruption strains were confirmed by PCR using primer sets annealing outside of the 5′- and 3′- homologous regions for homologous recombination (outside pair, indicated by gray arrowheads) and the 5′- and 3′-terminal regions of the coding region of the target genes (inside pair, indicated by black arrowheads). The amplified DNA fragments were analyzed by agarose electrophoresis and ethidium bromide staining. Lanes 1 and 2, outside; lanes 3 and 4, inside: Lanes 1 and 3, KPD1; lanes 2 and 4, each disruption strain (left, KPD1ΔTK2200 (Δ*arcE*); center, KPD1ΔTK0871 (Δ*arcB*); right, KPD1ΔTK2158 (Δ*arcC*)). DM3100 ExcelBand 1 KB DNA Ladder (SMOBIO Technology, Taiwan, China) was used as a DNA marker (M). **(B)** The *T. kodakarensis*-*E. coli* shuttle vector Pemp contains a replication initiator (rep74) from pLC64 (gray) and gene cassettes of pyruvoyl-dependent arginine decarboxylase (*pdaD*, red) from *Pyrococcus furiosus* and orotidine-5′-phosphate decarboxylase (*pyrF*, blue) from *T. kodakarensis*. Pcps additionally contains CPS genes from *Pyrococcus chitonophagus* (CHITON_1945 and CHITON_1946, purple) downstream of a constitutive promoter of the cell surface glycoprotein gene (TK0895, cyan) from *T. kodakarensis.* Pemp derivatives ParcE and ParcB contain TK2200 (orange) and TK0871 (green), respectively, from *T. kodakarensis* with their respective native promoter regions (yellow and light green).

To investigate the role of each gene in CP biosynthesis, the *arcE* (TK2200), *arcB* (TK0871), and *arcC* (TK2158) genes were individually disrupted in the host strain *T. kodakarensis* KPD1 (Figure 1) (Shimosaka et al., 2019). *T. kodakarensis* KPD1 is a strain deleted of the *pyrF* gene, encoding orotidine 5′-phosphate decarboxylase, and the *pdaD* gene, encoding pyruvoyl-dependent arginine decarboxylase. Orotidine 5′-phosphate decarboxylase catalyzes the final reaction in the biosynthesis of UMP (Supplementary Figure 1B). *T. kodakarensis* strains disrupted of *pyrF* can no longer synthesize UMP, however, they can grow when uracil is supplemented in the medium through the function of uracil phosphoribosyltransferase. On the other hand, the arginine decarboxylase reaction is responsible for the generation of agmatine, which is necessary for the synthesis of putrescine and other polyamines in *T. kodakarensis*. *T. kodakarensis* strains disrupted of *pdaD* cannot grow without the addition of agmatine to the medium. Therefore, KPD1 displays auxotrophy for both uracil and agmatine. Gene disruption of *arcE*, *arcB* and *arcC* using KPD1 as the host strain resulted in the isolation of the mutant strains Δ*arcE*, Δ*arcB*, and Δ*arcC*, respectively (Figure 2A). As this study addresses metabolism related to pyrimidine biosynthesis, which involves *pyrF*, we could not utilize *T. kodakarensis* strains with disruptions in the *pyrF* gene (Supplementary Figure 1B). To enable functional evaluation of pyrimidine biosynthesis, we introduced an autonomously replicating plasmid, Pemp (Figure 2B), into each strain. This plasmid carries expression cassettes for *pyrF* from *T. kodakarensis* and pyruvoyl-dependent arginine decarboxylase gene *pdaD* from *P. furiosus*. The resulting strains, which all possess functional *pyrF* and *pdaD* genes, were designated KPD1-Pemp, Δ*arcE*-Pemp, Δ*arcB*-Pemp, and Δ*arcC*-Pemp, respectively.

**Figure 3:**
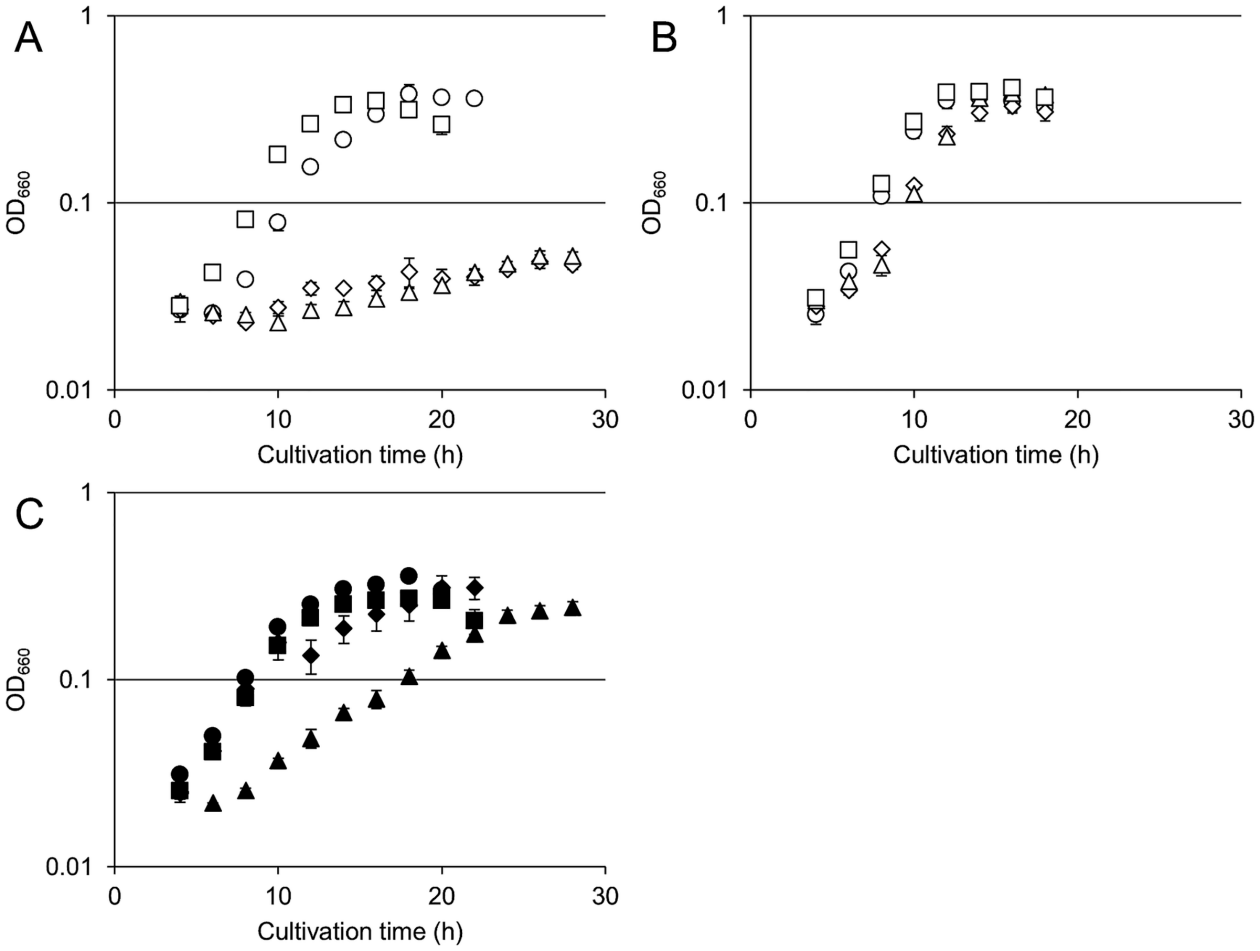
Growth characteristics of *T. kodakarensis* strains with or without pyrimidines. **(A)** Growth of each strain was evaluated in the synthetic amino acid medium ASW-AA-m1-S^0^-Pyr, which lacks pyrimidine compounds. Symbols: KPD1-Pemp (open circles), Δ*arcE*-Pemp (open diamonds), Δ*arcB*-Pemp (open triangles), Δ*arcC*-Pemp (open squares). **(B)** Growth of each strain was evaluated in the synthetic amino acid medium ASW-AA-m1-S^0^-Pyr-Ura, which contains 5 mg L^−1^ uracil. Symbols: KPD1-Pemp (open circles), Δ*arcE*-Pemp (open diamonds), Δ*arcB*-Pemp (open triangles), Δ*arcC*-Pemp (open squares). **(C)** Growth of each strain harboring a carbamoyl phosphate synthetase expression plasmid was evaluated in ASW-AA-m1-S^0^-Pyr. Symbols: KPD1-Pcps (closed circles), Δ*arcE*-Pcps (closed diamonds), Δ*arcB*-Pcps (closed triangles), Δ*arcC*-Pcps (closed squares). Error bars indicate the SD values of three independent culture experiments.

The growth of each strain was evaluated in a defined synthetic amino acid medium ASW-AA-m1-S^0^-Pyr, which lacks pyrimidine compounds. Under these conditions, the control strain KPD1-Pemp and the *arcC* disruption strain Δ*arcC*-Pemp reached stationary phase within 16 to 18 h, with final optical densities (OD_660_) of 0.38 and 0.35, respectively (Figure 3A). Each specific growth rate *μ* was calculated as 0.34 and 0.36 h^−1^. In contrast, no detectable growth was observed for the *arcE* and *arcB* disruption strains (Δ*arcE*-Pemp and Δ*arcB*-Pemp). When 5 mg/L of uracil was added to the medium (ASW-AA-m1-S^0^-Pyr-Ura), growth was restored in all strains (Figure 3B). KPD1-Pemp and Δ*arcC*-Pemp reached stationary phase after approximately 12 h with *μ* values of 0.43 and 0.39, while Δ*arcE*-Pemp and Δ*arcB*-Pemp reached stationary phase after approximately 14 h with *μ* of 0.35 and 0.39. The *arcE* and *arcB* genes were re-introduced into the Δ*arcE* and Δ*arcB* strains, resulting in Δ*arcE*-ParcE and Δ*arcB*-ParcB, respectively. Δ*arcE*-ParcE and Δ*arcB*-ParcB no longer displayed auxotrophy towards uracil (Supplementary Figure 3), indicating that the uracil auxotrophy observed in the disruption strains was due solely to the lack of *arcE* or *arcB* and not due to polar effects brought about by disrupting the respective loci. The results demonstrate that *arcE* and *arcB* are essential for *de novo* pyrimidine biosynthesis in *T. kodakarensis*, whereas the endogenous *arcC* (carbamate kinase) is not sufficient to compensate for the loss of ArcE/ArcB under these conditions.

### 2.3 Introduction of CPS from *Pyrococcus chitonophagus* restores growth without pyrimidines in *arcE* and *arcB* disruption strains

To determine whether the pyrimidine auxotrophy observed in the *arcE* and *arcB* disruption strains was due to a deficiency in intracellular CP, we attempted to restore the supply of CP. As CP is thermolabile and highly polar, we assumed it highly unlikely that CP would be efficiently taken up by the cell, or remain stable in the medium under the high-temperature culture conditions of *T. kodakarensis*. We thus introduced an internal CP-producing system that is mechanistically distinct from the native ArcE/ArcB or ArcC. Specifically, we constructed a plasmid expressing the genes encoding small and large subunits of carbamoyl phosphate synthetase from a Thermococcales archaeon. Homologs of the canonical CPS are present on the genome of *P. chitonophagus*: CHITON_1946 (35.7% identical to *E. coli* CPS small subunit *carA*) and CHITON_1945 (40.7% identical to *E. coli* CPS small subunit *carB*). The two genes were placed under the control of the strong, constitutive promoter of the cell surface glycoprotein gene from *T. kodakarensis* (*csg*, TK0895) (Figure 2B). The plasmid, Pcps, was introduced into the host and gene-disruption strains, generating KPD1-Pcps, Δ*arcE*-Pcps, Δ*arcB*-Pcps, and Δ*arcC*-Pcps.

When cultured in pyrimidine-free medium (ASW-AA-m1-S^0^-Pyr), the control strain KPD1-Pcps and the *arcC* disruptant Δ*arcC*-Pcps reached stationary phase within 18 h, with maximum OD_660_ values of 0.36 and 0.27, respectively (Figure 3C). Both *μ* were 0.33 h^−1^. Although the *arcE* and *arcB* disruption strains harboring Pemp did not grow in this medium, the respective strains harboring Pcps displayed growth. Δ*arcE*-Pcps reached stationary phase at approximately 20 h with an OD_660_ value of 0.31 and *μ* of 0.32 h^−1^. Δ*arcB*-Pcps displayed slower growth (*μ* = 0.13 h^−1^) and reached the stationary phase at approximately 28 h with an OD_660_ value of 0.24. These findings suggest that the pyrimidine auxotrophy observed in the *arcE* and *arcB* disruption was primarily due to loss of CP supply through the native ArcE/ArcB, and that this could be partially complemented by heterologous expression of *P. chitonophagus* CPS in the disruption strains.

From a different perspective, the differences in growth between Δ*arcB*-Pemp and Δ*arcB*-Pcps provide information on how CP is synthesized in different members of Thermococcales. Δ*arcB*-Pemp harbors an intact *arcC* on its genome and should have an active CK. Nevertheless, this strain cannot grow when *arcB* is disrupted, suggesting that CK cannot provide, at least to levels that can support growth, sufficient amounts of CP under these conditions. On the other hand, Δ*arcB*-Pcps displayed growth under the same conditions, indicating that the canonical CPS homolog provides CP at levels sufficient to support growth. The results suggest that in members of Thermococcales that harbor CPS homologs, CP is synthesized from bicarbonate and ammonia through the function of CPS rather than CK. In organisms such as *T. kodakarensis*, which lack CPS homologs, CP must instead be supplied via the arginine degradation mediated by ArcE and ArcB.

### 2.4 Contribution of the arginine synthetase pathway to [NiFe] hydrogenase-dependent hydrogen production

As the source of the cyanide ligands in the active center of [NiFe] hydrogenase have only been studied *in vivo* in *E. coli*, we examined whether the arginine synthetase pathway also contributes to [NiFe] hydrogenase maturation by providing CP in *T. kodakarensis*. *T. kodakarensis* harbors two [NiFe]-hydrogenases: a membrane-bound hydrogenase (Mbh), and a cytosolic hydrogenase (Hyh) (Kanai et al., 2011). It has previously been demonstrated that *T. kodakarensis* requires the membrane-bound [NiFe] hydrogenase Mbh for growth when sodium pyruvate or maltodextrin is supplied in medium composed of artificial seawater, yeast extract and tryptone (Kanai et al., 2011). When sodium pyruvate/maltodextrin is replaced with elemental sulfur (S^0^), S^0^ is used as the terminal electron acceptor generating hydrogen sulfide, a mode of growth that does not require the activity of [NiFe] hydrogenase. It can thus be expected that strains without an active Mbh cannot grow on pyruvate or maltodextrin, but can grow in medium with S^0^. Here we applied ASW-YT-m1-Pyr or ASW-YT-m1-Mdx medium as a hydrogenase-dependent growth condition, and ASW-YT-m1-S^0^ medium as a hydrogenase-independent growth condition.

**Figure 4:**
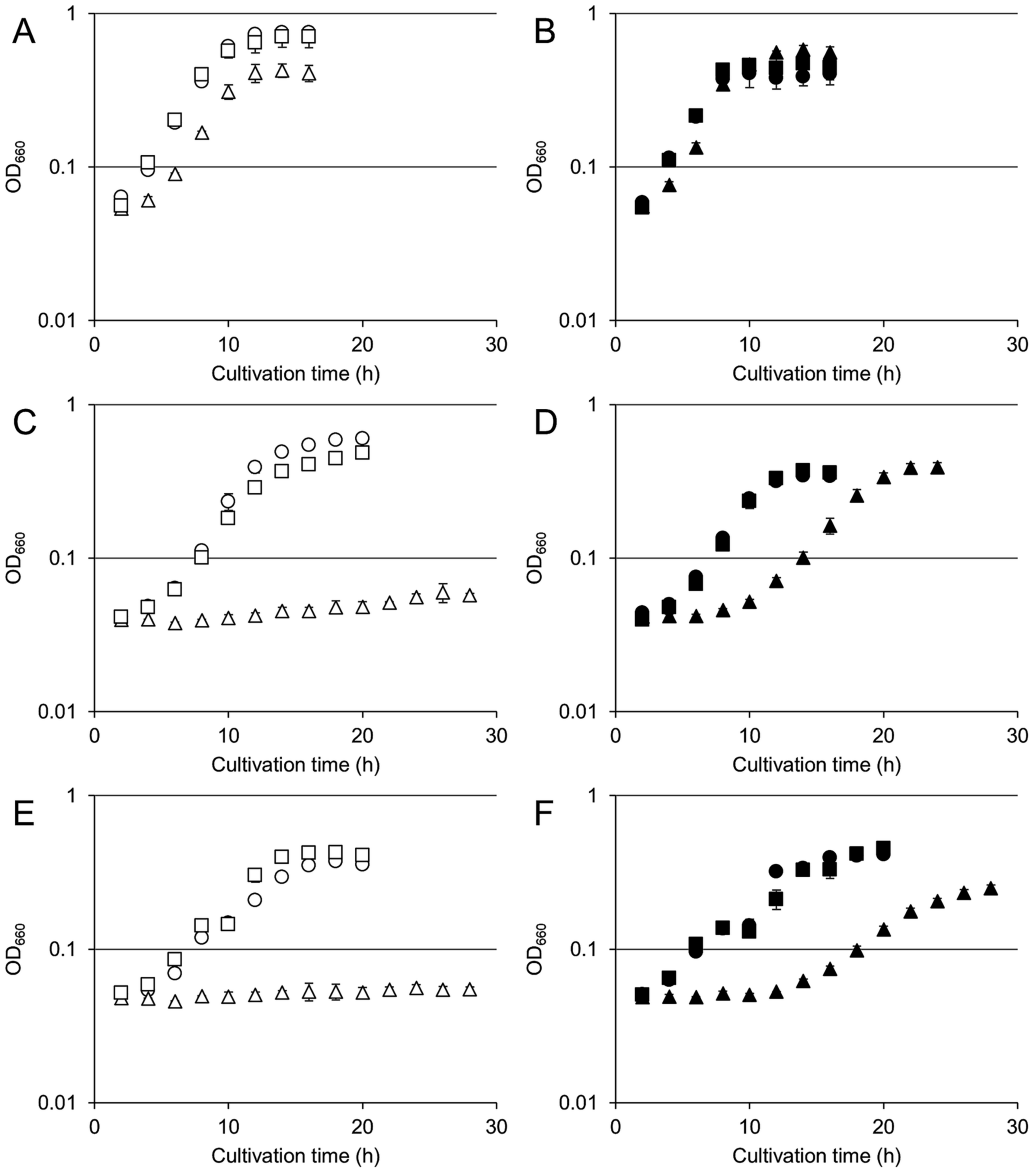
Growth characteristics of *T. kodakarensis* strains under hydrogenase-independent or hydrogenase-dependent conditions. Growth of each strain was evaluated under hydrogenase-independent condition (ASW-YT-m1-S^0^, **A,B**), or hydrogenase-dependent conditions (ASW-YT-m1-Pyr, **C,D**; ASW-YT-m1-Mdx, **E,F**). Symbols: KPD1-Pemp (open circles), Δ*arcB*-Pemp (open triangles), Δ*arcC*-Pemp (open squares), KPD1-Pcps (closed circles), Δ*arcB*-Pcps (closed triangles), Δ*arcC*-Pcps (closed squares). Error bars indicate the SD values of three independent culture experiments.

Growth of the host strain and the *arcE*, *arcB* and *arcC* disruption strains were evaluated under hydrogenase-dependent and -independent conditions. The results of the *arcE* disruption strain are described below. In medium in which hydrogenase is not essential (ASW-YT-m1-S^0^), the host strain and the *arcB* and *arcC* disruption strains transformed with the empty plasmid Pemp displayed growth, reaching stationary phase in approximately 14 h with *μ* of 0.33–0.34 h^−1^ (Figure 4A). The *arcB* disruption strain Δ*arcB-*Pemp displayed a slight impairment in growth (*μ* = 0.31 h^−1^) with an extended lag phase and lower cell density at the stationary phase. Under hydrogenase-dependent conditions (ASW-YT-m1-Pyr and ASW-YT-m1-Mdx), more dramatic differences in growth were observed (Figures 4C,E). The host strain KPD1*-*Pemp and the *arcC* disruption strain Δ*arcC-*Pemp displayed robust growth, indicating that the strains harbored an active hydrogenase. In contrast, Δ*arcB-*Pemp showed no detectable growth under these conditions, suggesting that the strain did not harbor hydrogenase activity.

In terms of growth of the *arcE* disruption strain, it has been reported that citrulline is present in yeast extract at levels comparable to those of amino acids (Gancedo and Gancedo, 1973). Numerous studies also report that citrulline is present in casein or its tryptic digests (Wada, 1933; Tapal et al., 2016; Li et al., 2020). Since citrulline is the product of the ArcE reaction, it would be difficult to genetically evaluate the function of ArcE in medium with yeast extract or tryptone. Citrulline included in the yeast extract and tryptone could be utilized to provide CP through the ArcB reaction, regardless of the presence or absence of ArcE. Indeed, the Δ*arcE-*Pemp strain displayed growth in the hydrogenase-dependent medium ASW-YT-m1-Pyr (Supplementary Figure 4). When Δ*arcE-*Pemp was grown in medium with tryptone alone and without yeast extract (ASW-T-m1-Pyr), Δ*arcE-*Pemp displayed a delay in growth compared to the host strain KPD1*-*Pemp, suggesting that the supply of citrulline from tryptone was rate-limiting. In principle, evaluation of growth in a synthetic medium dependent on hydrogenase would provide a condition without extracellular citrulline. However, *T. kodakarensis* cannot grow in synthetic medium when pyruvate or maltodextrin is added but S^0^ is absent. It is thus not possible to test a growth condition based on defined media and dependent on hydrogenase, as the addition of S^0^ is required for growth in synthetic media.

To determine whether the lack of growth of Δ*arcB-*Pemp in ASW-YT-m1-Pyr and ASW-YT-m1-Mdx (Figures 4C,E) resulted from a deficiency in CP biosynthesis, we evaluated the host strain and the *arcB* or *arcC* disruption strains harboring the CPS expression plasmid Pcps (Figures 4B,D,F). In ASW-YT-m1-S^0^ medium, in which hydrogenase is not essential (Figure 4B), all strains again displayed growth. Notably, the partial impairment of growth observed for the *arcB* disruption strain (Figure 4A) was no longer observed in the strain transformed with the CPS expression plasmid. This suggests that the impairment in growth was due to an insufficient supply of CP in the *arcB* disruption strain. Under hydrogenase-dependent conditions, although initiation of growth was delayed, the *arcB* disruption strain harboring the CPS expression plasmid ultimately reached comparable cell density stationary phase after approximately 22–28 h (Figures 4D,F). The partial restoration of growth indicates that heterologous expression of CPS can compensate for the loss of ArcB, at least to some extent, in supporting hydrogenase maturation. We also evaluated the effects of introducing CPS into the *arcE* disruption strain. The delay in growth of the Δ*arcE*-Pemp strain in ASW-T-m1-Pyr was clearly reduced in Δ*arcE*-Pcps (Supplementary Figure 4D), suggesting that the delay in growth was due to insufficient supply of CP. The result also implies that the ArcE reaction partially contributed to CP generation from arginine in cells grown in ASW-T-m1-Pyr medium.

Taken together, the results indicate that CP is the precursor of the cyanide ligands in the active site of [NiFe] hydrogenase and that it is provided by the arginine synthetase pathway in *T. kodakarensis*.

### 2.5 Distribution of genes related to CP metabolism in Thermococcales

Comparative genomic analysis revealed that genes for pyrimidine biosynthesis and [NiFe] hydrogenase maturation genes are conserved across Thermococcales, whereas canonical CPS genes are present in only 8 of 42 species registered in KEGG (Kanehisa and Goto, 2000; Kanehisa et al., 2024) (Supplementary Table 1). In contrast, the arginine synthetase pathway genes, *arcE* and *arcB*, are conserved throughout this order, indicating that most members of Thermococcales rely on this pathway as the primary source of CP. In species which harbor CPS, the presence of CPS is tightly linked with the presence of argininosuccinate synthetase and argininosuccinate lyase genes, which are involved in canonical arginine biosynthesis. This co-occurrence strongly suggests that CP produced by CPS is also used for the production of arginine.

## 3 Discussion

The gene disruption/complementation and phenotype analyses in this study demonstrated that the arginine synthetase pathway is crucial for CP biosynthesis in *T. kodakarensis*. The disruption of *arcE* or *arcB* resulted in pyrimidine auxotrophy, and the *arcB* disruption additionally impaired growth under hydrogenase-dependent energy metabolism. Both phenotypes were partially restored by heterologous expression of the CPS gene from *P. chitonophagus*. This confirms that *T. kodakarensis*, lacking canonical CPS genes, relies on the arginine degradation pathway mediated by ArcE and ArcB for the production of CP, which is utilized for both anabolic and bioenergetic processes. Compared to *arcE* disruption strains, the degree of growth recovery of Δ*arcB* strains following recombinant CPS introduction was not complete. When cells simultaneously express CK and CPS, the CP produced by CPS consuming two molecules of ATP is broken down by CK while recovering only one molecule of ATP. The absence of ArcB may be amplifying the effects of this potential futile cycle by not allowing the CP produced by CPS to be converted to citrulline, resulting in increased degradation by CK.

The results in this study provide evidence on the *de novo* synthesis of carbamoyl phosphate in Thermococcales. We observed that the *arcC* disruption strain did not display the same growth defects as the *arcE* and *arcB* mutants. While CK has been proposed as a potential candidate for CPS in some archaea such as *P. furiosus* (Durbecq et al., 1997), our results suggest that in *T. kodakarensis*, CK cannot compensate for the loss of ArcE/ArcB whereas introduction of a CPS partially restored growth. These findings suggest that the canonical CPS, and not CK, is responsible for CP biosynthesis in organisms that harbor CPS, including *P. furiosus* and *P. chitonophagus* (Supplementary Table 1). This highlights the importance of CP biosynthesis in organisms that lack canonical CPS homologs, such as *T. kodakarensis* and *P. abyssi*. Our results suggest that in *T. kodakarensis*, at least under the growth condition applied, CP synthesis relies solely on the arginine synthetase pathway. For further elucidation of CP biosynthesis strategies within Thermococcales members harboring CPS, functional analyses of mutants disrupted of the ArcB and/or CPS genes in species such as *P. chitonophagus* or *P. furiosus* will be necessary.

Under hydrogenase-dependent conditions, the *arcB* disruption strain failed to grow, confirming the essential role of ArcB in CP production for hydrogenase maturation. In contrast, strains expressing heterologous CPS from *P. chitonophagus* partially restored growth in the *arcB* disruption strain. As the supply of CP is necessary for the maturation of [NiFe] hydrogenases, this suggests that CP is the precursor for the cyanide ligands of the [NiFe] hydrogenase in this archaeon, which has previously only been demonstrated in bacteria (Paschos et al., 2001; Reissmann et al., 2003).

As most Thermococcales members do not harbor CPS, argininosuccinate synthetase and argininosuccinate lyase genes (Supplementary Table 1), we presume that these members are auxotrophs for arginine, as was demonstrated in the case of *T. kodakarensis* (Michimori et al., 2024). In addition, CP for pyrimidine biosynthesis and [NiFe] hydrogenase maturation in these organisms must be supplied via ArcE/ArcB. These observations support a model in which most Thermococcales are auxotrophs for arginine and rely on the arginine synthetase pathway for CP generation, while CPS is retained only in a subset of species to support canonical arginine biosynthesis.

**Figure 5:**
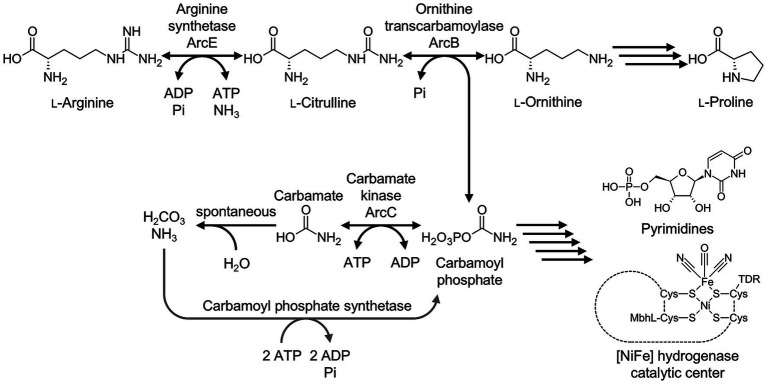
The arginine synthetase pathway and carbamoyl phosphate metabolism in *T. kodakarensis*. *T. kodakarensis* degrades arginine via the energy conserving reaction catalyzed by arginine synthetase and ornithine transcarbamoylase to obtain ornithine and carbamoyl phosphate. Ornithine is a precursor for proline biosynthesis. Carbamoyl phosphate is utilized not only for ATP generation via carbamate kinase activity, but also for pyrimidine nucleotide biosynthesis and maturation of the catalytic center of the [NiFe] hydrogenase, Mbh. *T. kodakarensis* does not harbor a carbamoyl phosphate synthetase (indicated by a gray arrow), however, heterologously expressed carbamoyl phosphate synthetase from *Pyrococcus chitonophagus* can complement the role of carbamoyl phosphate production in *arcE* or *arcB* disruption mutants. MbhL, large subunit of Mbh; TDR represents the three amino acid residues (Thr-Asp-Arg) at the C-terminus of mature MbhL.

This study provides new insights into the roles of the arginine synthetase pathway in CP biosynthesis in *T. kodakarensis*, particularly in the context of pyrimidine biosynthesis and hydrogenase maturation. Taken together with previous knowledge, arginine can thus be considered a primary substrate of Thermococcus species, acting not only as a catabolic substrate, but also providing precursor compounds for proline, polyamines, pyrimidine nucleotides and the active center of hydrogenase (Figure 5). This study also represents the first report demonstrating *in vivo* that CP serves as a substrate for pyrimidine biosynthesis and [NiFe] hydrogenase maturation in Archaea.

## 4 Materials and methods

### 4.1 Chemicals, strains and culture conditions

Unless mentioned otherwise, chemicals were purchased from Fujifilm Wako Pure Chemicals (Osaka, Japan) or Nacalai Tesque (Kyoto, Japan), or MilliporeSigma (Burlington, MA, USA). *Escherichia coli* DH5*α* and *E. coli* HST08 (TaKaRa, Ohtsu, Japan) were cultivated at 37 °C in Lysogeny Broth (LB) medium containing 100 mg L^−1^ ampicillin and used for plasmid construction. *Thermococcus kodakarensis* KOD1 (Morikawa et al., 1994; Atomi et al., 2004), KU216 (Sato et al., 2005), and KPD1 (Shimosaka et al., 2019) and its derivative strains and *Pyrococcus chitonophagus* GC74 (Horiuchi et al., 2016) were cultivated anaerobically at 85 °C in following media: ASW-AA-m1-S^0^-Pyr (Su et al., 2023), ASW-YT-m1-S^0^ (Su et al., 2023), ASW-YT-m1-Pyr (elemental sulfur in ASW-YT-m1-S^0^ was replaced with 5 g L^−1^ sodium pyruvate), and ASW-YT-m1-Mdx (elemental sulfur in ASW-YT-m1-S^0^ was replaced with 5 g L^−1^ maltodextrin (Amycol 3-L; Nippon Starch Chemical, Osaka, Japan)). Yeast extract and tryptone used in this study were purchased from Nacalai Tesque. When required, 5 mg L^−1^ uracil or 1 mM agmatine sulfate was added to the medium. Optical density at 660 nm (OD_660_) was measured using an Ultrospec 7,500 (Biochrom, Cambridge, UK). All growth assays were conducted in triplicate.

### 4.2 Plasmid construction

Plasmids for disrupting TK2200, TK0871, and TK2158 were constructed from pUD3 carrying the *pyrF* marker cassette. Approximately 1,000-bp upstream and downstream homologous regions of TK2200 locus were amplified from the KU216ΔTK2200 strain (Kanai et al., 2013; Michimori et al., 2024) using primers dTK2200F1/dTK2200R1 and inserted into HincII-digested pUD3 (pΔTK2200). 1,000-bp upstream and downstream homologous regions of TK0871 and TK2158 were amplified from the KU216 genome using primers dTK0871F1/dTK0871R1 and dTK2158F1/dTK2158R1 and inserted into the HincII site of pUD3 (prepΔTK0871 and prepΔTK2158, respectively). The region containing the ORF was removed from prepΔTK0871 and prepΔTK2158 by inverse PCR using primers dTK0871F2/dTK0871R2 and dTK2158F2/dTK2158R2 followed by self-ligation (pΔTK0871, pΔTK2158). The resulting pΔTK2158 also lacked the base just before the start codon, but this is unlikely to affect the surrounding genes.

The shuttle vector Pemp, which contains the *pyrF* and *pdaD* expression cassettes under constitutive promoters, was used for plasmid-based expression in *T. kodakarensis*. pRPETK1697 (Shimosaka et al., 2019) was digested with EcoRI and SphI to obtain a backbone containing the pUC118-derived region, a replication initiator (rep74) from pLC64 (Santangelo et al., 2008), and a gene cassette of pyruvoyl-dependent arginine decarboxylase (*pdaD*) from *P. furiosus*. From *T. kodakarensis* KOD1 genomic DNA, we amplified the promoter region of TK0895 using primers Pemp-fA/Pemp-rA, the terminator region between TK1430 and TK1431 using Pemp-fB/Pemp-rB, the open reading frame of TK2276 (*pyrF*) using Pemp-fC/Pemp-rC, and the promoter region of TK2279 using Pemp-fD/Pemp-rD. These fragments were joined into a single DNA fragment by overlap extension PCR, digested with EcoRI and SphI, and inserted into the above backbone to generate Pemp. To construct Pcps, the CPS genes from *P. chitonophagus* (CHITON_1945 and CHITON_1946) were amplified using InfIns-Pcps-f/InfIns-Pcps-r. The genes were inserted downstream of the constitutive promoter of the cell surface glycoprotein gene (*csg*, TK0895) of *T. kodakarensis*, using in-fusion reaction. To construct ParcE and ParcB, the ArcE and ArcB genes (TK2200 and TK0871, respectively) with corresponding promoter regions were amplified from *T. kodakarensis* KOD1 genome using NExTK2200_F/ExTK2200_R or NExTK0871_F/ExTK0871_R. The fragments were ligated with linearized Pemp using Pemp_F/Pemp_Nex_R by in-fusion reaction.

All constructs were verified by DNA sequencing. All primer sequences for plasmid construction and sequence analysis are listed in Supplementary Tables 2, 3.

### 4.3 Gene disruption and strain construction

For gene disruption, homologous recombination was performed in *T. kodakarensis* KPD1 (Δ*pyrF* Δ*pdaD*) according to the previously reported method for a *pyrF*-deleted strain KU216 (Δ*pyrF*) as the host (Sato et al., 2003, 2005). pΔTK2200, pΔTK0871 or pΔTK2158 were introduced onto the genome of KPD1 via single cross-over insertion, and transformants harboring the *pyrF* gene carried by each plasmid were enriched in ASW-AA-m1-S^0^-Pyr media without pyrimidines. Recombinants whose *pyrF* gene was removed via a second recombination event were selected on solid ASW-YT-m1 plate supplemented with 7.5 g L^−1^ 5-fluoroorotic acid for the counterselection (Su et al., 2023). Deletions were confirmed by colony PCR and sequencing.

For gene introduction or complementation, strains harboring Pemp, Pcps, ParcE or ParcB plasmids were constructed by transforming each gene-disrupted strain with the respective plasmid. Positive clones were selected using the *pdaD* marker.

### 4.4 Growth assays under pyrimidine-depleted or hydrogenase-dependent conditions

To assess the role of *arcE*, *arcB*, and *arcC* in *de novo* pyrimidine biosynthesis, strains carrying either Pemp, Pcps, ParcE or ParcB were cultivated in 10 mL ASW-AA-m1-S^0^-Pyr medium with/without uracil supplementation. To determine whether the arginine synthetase pathway contributes to hydrogenase maturation, strains were cultured under hydrogenase-dependent (10 mL ASW-YT-m1-Pyr or ASW-YT-m1-Mdx) and hydrogenase-independent (10 mL ASW-YT-m1-S^0^) conditions. Optical density at 660 nm (OD_660_) was measured using an Ultrospec 7,500 (Biochrom, Cambridge, UK). All growth assays were conducted in triplicate.

### 4.5 Gene distribution analysis

The distributions of genes related to CP metabolism (arginine synthetase, *arcE*; ornithine transcarbamoylase, *arcB*; carbamate kinase, *arcC*; CPS small subunit, *carA*; CPS large subunit, *carB*; argininosuccinate synthase, *argG*; argininosuccinate lyase, *argH*; hydrogenase maturation proteins, *hypABCDEF*; aspartate carbamoyltransferase regulatory subunit, *pyrI*; aspartate carbamoyltransferase catalytic subunit, *pyrB*; dihydroorotase, *pyrC*; dihydroorotate dehydrogenase electron transfer subunit, *pyrDII*; dihydroorotate dehydrogenase catalytic subunit, *pyrDI*; orotate phosphoribosyltransferase, *pyrE*; orotidine-5′-phosphate decarboxylase, *pyrF*) in Thermococcales was surveyed in genome sequences from KEGG. Homologs were identified by BLASTP using representative protein from *T. kodakarensis* and *P. furiosus* as a query with thresholds of *E*-value ≤ 10^−50^ for *arcB*, *carA*, *hypB*, *hypE*, *pyrB* and *pyrDII*, *E*-value ≤ 10^−20^ for *hypC* and *hypF*, or *E*-value ≤ 10^−10^ for the others.

## Data Availability

The original contributions presented in the study are included in the article/supplementary material, further inquiries can be directed to the corresponding author.
