# Supplementary material for: The arginine synthetase pathway is responsible for carbamoyl phosphate synthesis in Thermococcus kodakarensis
Source: Front Microbiol. 2026 May 29;17:1761990. doi: 10.3389/fmicb.2026.1761990 (PMC13260591; doi:10.3389/fmicb.2026.1761990)
Supplement: Supplementary file 2 [file Data_Sheet_1.pdf]

Supplementary Material

1 Supplementary Tables and Figures

1.1 Supplementary Tables

1.1.1 Supplementary Table 1. Distribution of genes related to arginine synthetase pathway in Thermococcales. (A separate file "SupplementaryTable1.xlsx")

1.1.2 Supplementary Table 2. List of primers used for gene disruption in this study.

Use	Gene	Type	Primer name	5'-sequence-3'
For disruption vector construction	TK2200	A	dTK2200F1	GCCGTCCTCATCAACGAT
			dTK2200R1	CAAGTTCTCACACGCCCC
	TK0871	A	dTK0871F1	AAAACCAAAGGTTTCTAACCCTCAAAAAGCG
			dTK0871R1	CTGTCCGGGTAGAAGCGCC
		B	dTK0871F2	TTTTGGACTCTTTTCTCCATTTGTCTTTG
			dTK0871R2	GTGCATCACCGTATAGAACTGTGGA
	TK2158	A	dTK2158F1	GAGCGGGCGCGGGTG
			dTK2158R1	CTTGTGCTTCTCGTCGTACTCCCTGAT
		B	dTK2158F2	ATTTGGAGCAAAGTTTTTTGTTTTACCATTATTCTGTCTCAATTT
			dTK2158R2	GCTCTCACCTCCAGGGATGACTAATTACGC
For sequencing	TK2200	C	dTK2200seqF	CGATAAGCGGTAAATTTGGGAAGGATTCTAATGTAGC
			dTK2200seqR	TCCTGCCAGACCATGTATTCGTACTCCTTCTT
	TK0871	C	dTK0871seqF	CCCACTTGGAATGACAAAGGTCTCCTCAA
			dTK0871seqR	CGCCCAGCCTCGACAGATTGA
	TK2158	C	dTK2158seqF	ACCGTTGAGCTCCTCGGAAGGGA
			dTK2158seqR	CTCCTGTATCCTCTCGGGGGACATCTTG
	universal	D	M4S	CTGGCGAAAGGGGGATGTGC
			RVS	ACACTTTATGCTTCCGGCTC
For PCR analysis of gene disruptants	TK2200	E	dTK2200outF	CTGTTCCCCTGGTTGCTGACAT
			dTK2200outR	GAGCCTGCTCGGTGCGA
		F	dTK2200inF	ATGGCAGAGAAGAAAAAGAAGAGGGTTCTGATT
			dTK2200inR	TCACTTCTTGAGCTTCTCGCAGTTCTTGG
	TK0871	E	dTK0871outF	GAATATGAACCTAGGCACTTCAAATCTGACTTTTTGTC
			dTK0871outR	AAATAGACTGGATAAAGCTCGGAAAGTTCGACTC
		F	dTK0871inF	ATGGTGGTTAGCCTTGCAGGAAGAGATGT
			dTK0871inR	TCACACCTTAATCCCGCCCATGAC
	TK2158	E	dTK2158outF	CTGGCACACCTCCTAAAACTCCTCGGT
			dTK2158outR	CCTGACTACCCCAAGCCT
		F	dTK2158inF	ATGAAGAGAGTTGTCATAGCCTTGGGCG
			dTK2158inR	TCACTCCCGTACAACCTGCGTC

A: Primers for amplifying target genes and their 5'- and 3'-flanking regions.
B: Primers for inverse PCR amplifying 5'- and 3'-flanking regions and entire plasmid to exclude target genes.
C: Primers for sequencing 5'- or 3'-flanking region of target genes.
D: Primers annealing outside of multi cloning site of pUD3.
E: Primers annealing outside of homologous regions for homologous recombination.
F: Primers annealing within the target genes.

1.1.3 Supplementary Table 3. List of primers used for expression vector construction in this study.

Use	Target	Type	Primer name	5'-sequence-3'
For expression vector construction	TK0895 promoter	A	Pemp_fA	CCATGATTACGAATTCTATCGGCAAAAGGCGAATTATGTGTAGG
			Pemp_rA	GGATCCGCTAGCTAACTAGTGCTAGCTATCTAGAGCTAGCTACATATGACAACACCTCCTTGGGTTGTTGGGGCTTTAG
	TK1431 terminator	A	Pemp_fB	ATGTAGCTAGCTCTAGATAGCTAGCACTAGTTAGCTAGCGGATCCTTTCTTCTCCCTTTTCTCTTGTCC
			Pemp_rB	GAAATTCTGGAGGTGGTTTGAGTCGACACTTCTGTTTCGTTTTTATTTAG
	TK2276 ORF	A	Pemp_fC	CTAAATAAAAACGAAACAGAAGTGTCGACTCAAACCACCTCCAGAATTT
			Pemp_rC	GAGCGTTTGAGTCCTTCTGACGGCTCTTGGAGAGGGCCGTTAAAAAGGTGATGGCCATGGAGGAGAGCAGGCTCATTC
	TK2279 promoter	A	Pemp_fD	GAATGAGCCTGCTCTCCTCCATGGCCATCACCTTTTTAACGGCCCTC
			Pemp_rD	TGCCAAGCTTGCATGCCCGCAACGCGCATTTGCTCACCCG
	Pemp backbone for CPS expression	B	InfVec-Pcps-f	TTAGGGATCCTTTCTTCTCCCTTTTCTCTTGTCC
			InfVec-Pcps-r	CTCATATGACAACACCTCCTTGGGTTGTTGGGGC
	CHITON_1945 and CHITON_1946 ORF	B	InfIns-Pcps-f	GTGTTGTCATATGAGGGGTGAGGTAGGATAC
			InfIns-Pcps-r	AGAAAGGATCCCTAAACTCTACTTAGTGAA
	Pemp backbone for ArcE or ArcB expression	B	Pemp_F	TCCTTTCTTCTCCCTTTTCTCTTGTC
			Pemp_Nex_R	TTCGTAATCATGGTCATAGCTGTTTC
	TK2200 promoter and ORF	B	NExTK2200_F	GACCATGATTACGAAACTATACCCAATAGTCCACGGG
			ExTK2200_R	AGGGAGAAGAAAGGATCACTTCTTGAGCTTCTCGCAG
	TK0871 promoter and ORF	B	NExTK0871_F	GACCATGATTACGAACACCTGCAGTGAGATGTAGTACTTC
			ExTK0871_R	AGGGAGAAGAAAGGATCACACCTTAATCCCGCCCATG

A: Primers for amplification of target fragments and overlap extension PCR.
B: Primers for amplification of target fragments and in-fusion reaction.

1.2 Supplementary Figures

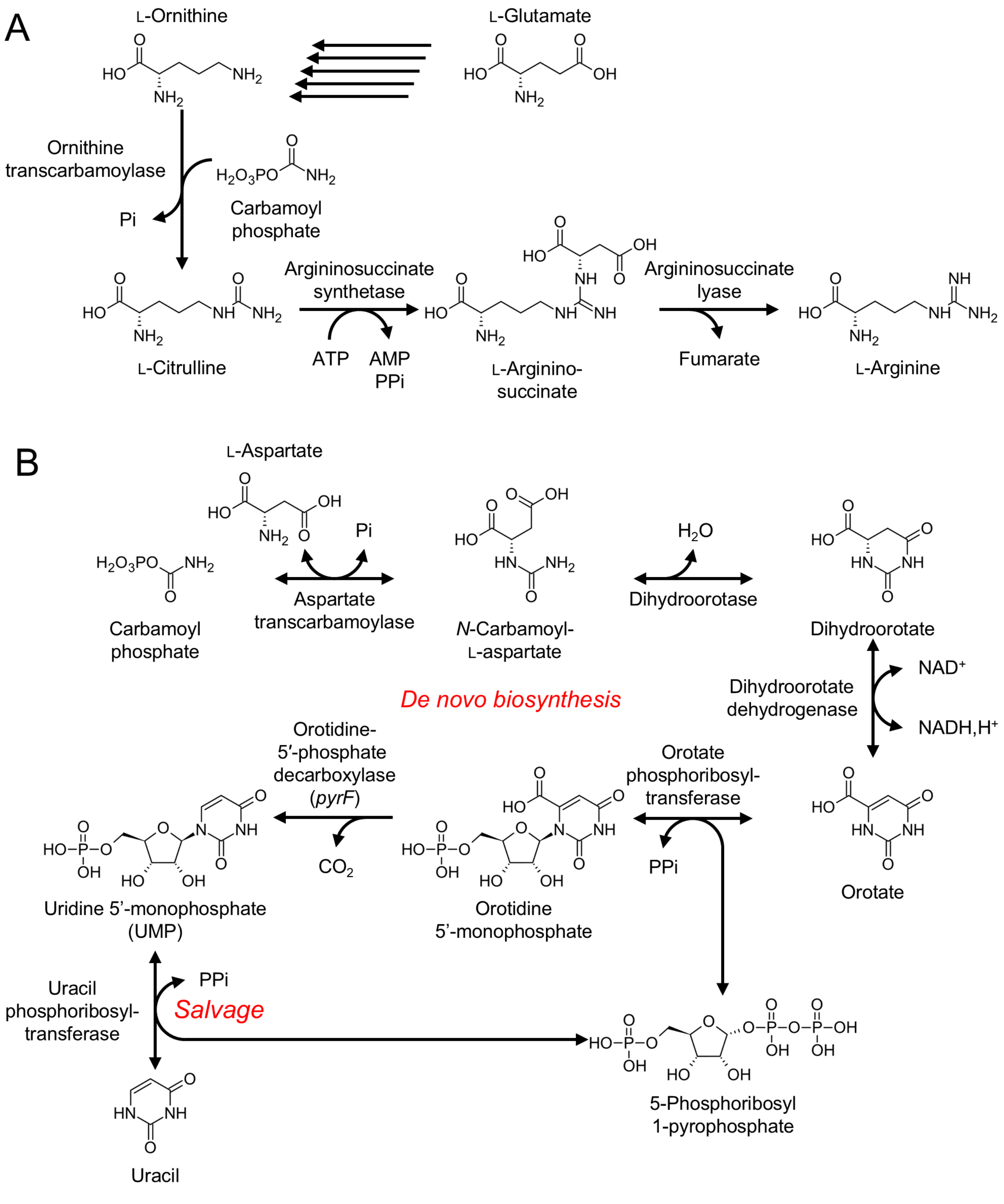

Supplementary Figure 1. Carbamoyl phosphate utilization in biosynthetic pathways. (***A***) Utilization of CP in arginine biosynthesis. CP serves as the carbamoyl donor for the formation of citrulline from ornithine, catalyzed by ornithine transcarbamoylase. Citrulline is then converted to argininosuccinate and subsequently to arginine. (***B***) Utilization of CP in pyrimidine biosynthesis. In the *de novo* pyrimidine biosynthesis pathway, CP reacts with aspartate to form carbamoyl aspartate, catalyzed by aspartate transcarbamoylase. Carbamoyl aspartate is cyclized to dihydroorotate, which is then oxidized to orotate. Orotate is converted to orotidine 5′-monophosphate by orotate phosphoribosyltransferase, and finally decarboxylated to uridine 5′-

monophosphate (UMP) by orotidine-5′-phosphate decarboxylase (encoded by *pyrF*). Through uracil phosphoribosyltransferase, UMP can also be produced by salvaging free uracil.

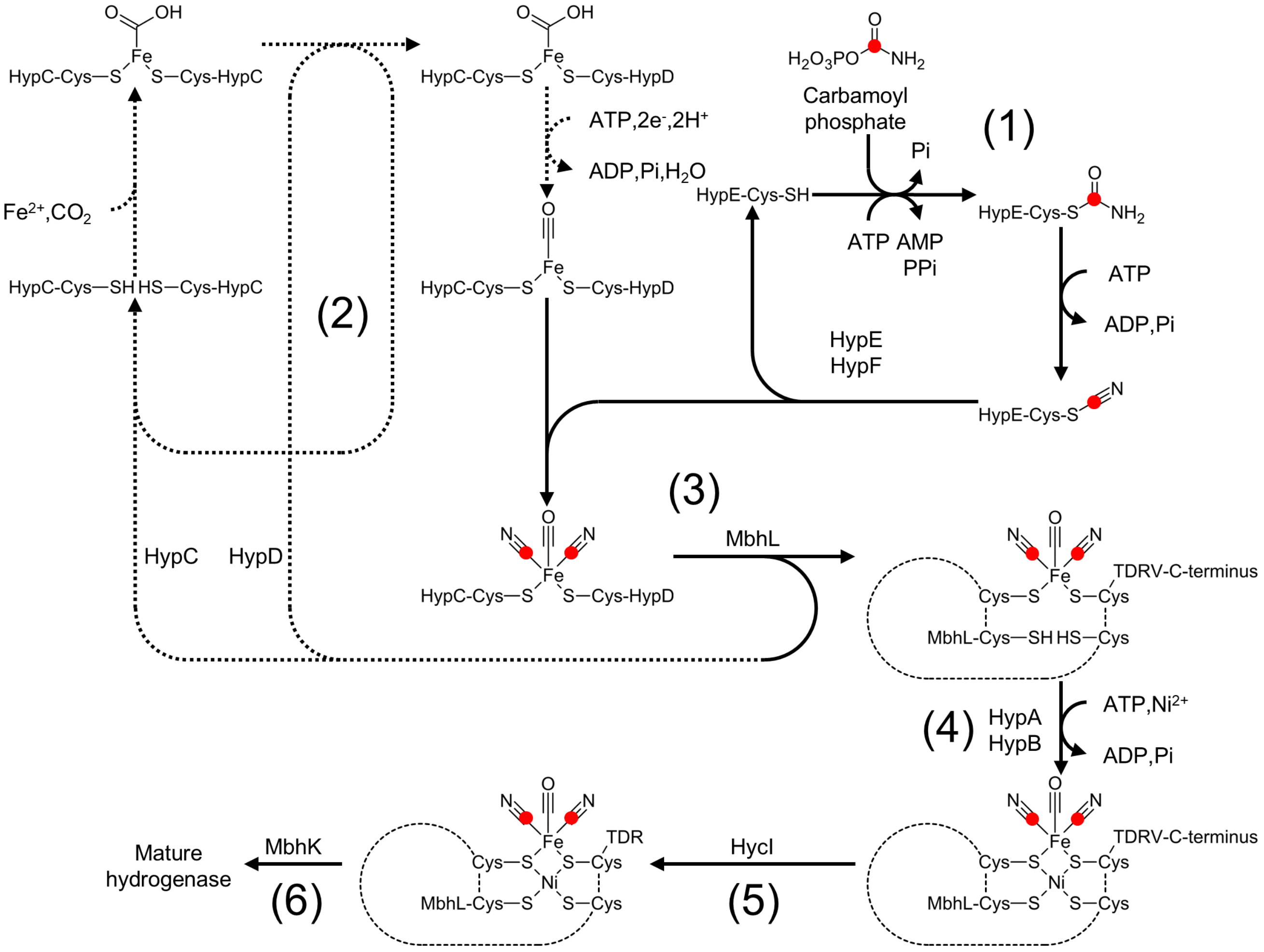

Supplementary Figure 2. Maturation of [NiFe]-hydrogenases: stepwise assembly of the $Fe(CN)_2CO$/Ni active site and processing of the large subunit. The [NiFe] active site is assembled in a stepwise manner involving HypA/B/C/D/E/F and dedicated endoproteases. (1) Cyanide ligand biosynthesis (from carbamoyl phosphate). CN^- ligands are derived from carbamoyl phosphate (CP) through the cooperative action of HypF and HypE. HypF activates CP using ATP, and transfers to the conserved C-terminal cysteine of HypE. HypE then catalyzes an ATP-dependent dehydration reaction, generating a thiocyanate-like intermediate, which ultimately yields CN^-. (2) CO ligand biosynthesis. In aerobes or microaerophiles, HypX has been shown to generate CO for the [NiFe] cofactor. In contrast, the source of CO in anaerobes is unclear. It has been proposed that HypCD itself could contribute to CO supply under anaerobic conditions, based on its redox-active [4Fe–4S] cluster and potential disulfide exchange chemistry. The pathway is indicated by dashed arrows. (3) Assembly of the $Fe(CN)_2CO$ fragment on HypCD. HypC and HypD form a scaffold complex where the $Fe(CN)_2CO$ fragment is assembled. Once formed, the fragment is transferred via HypC to the large subunit precursor. (4) Nickel insertion (HypA/HypB system). After the iron fragment is integrated, nickel is delivered by HypA and HypB. (5) Endoproteolytic processing of the large subunit. Following nickel insertion, specific maturation proteases cleave the C-terminal extension of the large subunit precursor. The cleavage site -ThrAspArgVal- is indicated as TDRV. This processing triggers structural rearrangements that stabilize the Cys-Fe-Ni coordination sphere, completing the catalytically competent active site. (6) Final assembly with the small subunit. The small subunit incorporates Fe-S clusters, then associates with the matured large subunit to form the active heterodimer. Carbon atoms derived from CP are indicated by red circles.

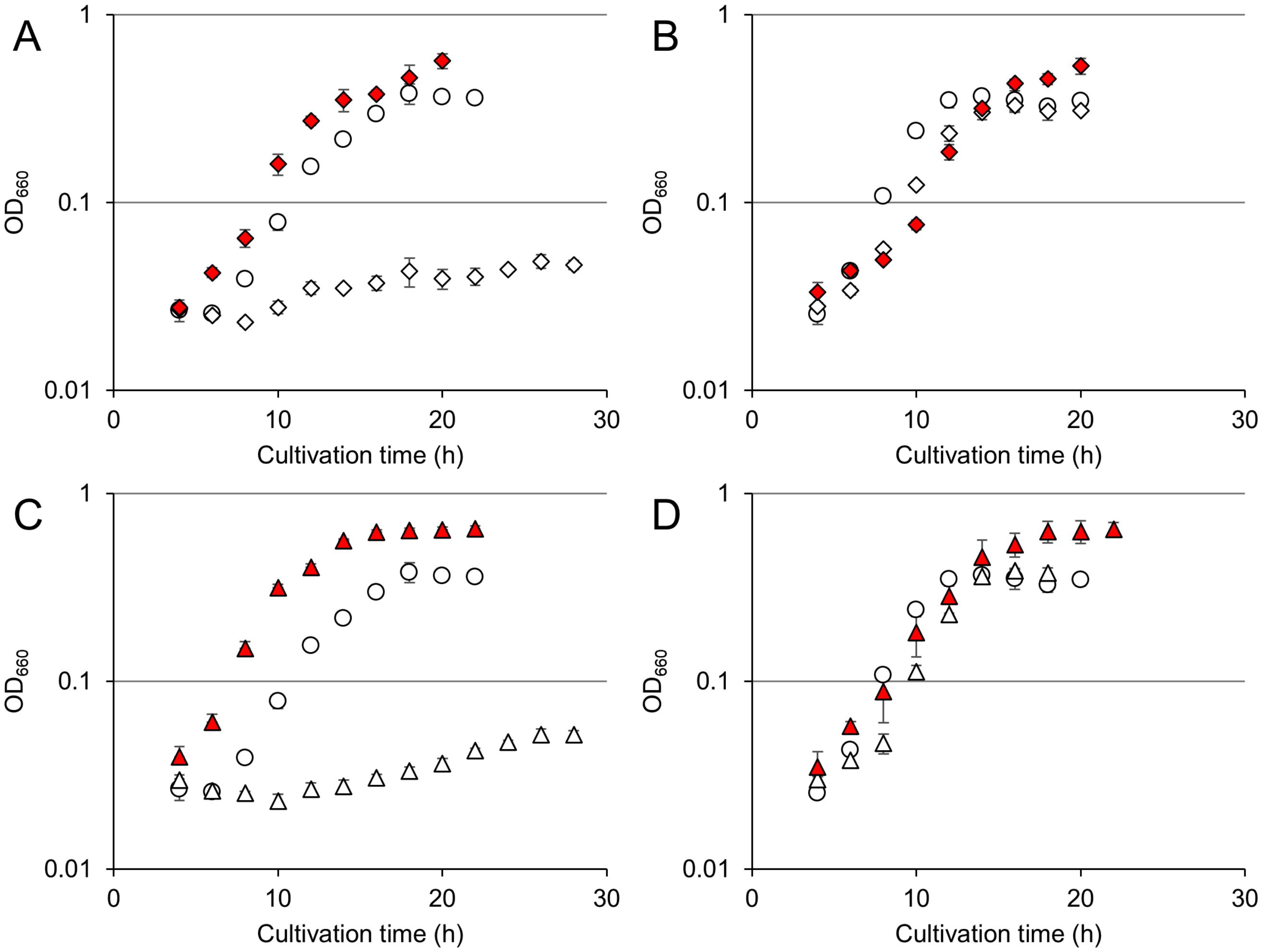

Supplementary Figure 3. Effects of *arcE* and *arcB* complementation on growth without pyrimidines. (***A***) The effect of *arcE* complementation on growth without pyrimidines was evaluated in the synthetic amino acid medium ASW-AA-m1-S^0-Pyr, which lacks pyrimidine compounds. Symbols: KPD1-Pemp (white circles), Δ*arcE*-Pemp (white diamonds), Δ*arcE*-ParcE (red diamonds). (***B***) Growth of each strain in the synthetic amino acid medium ASW-AA-m1-S^0-Pyr-Ura, which contains 5 mg/L uracil. Symbols: KPD1-Pemp (white circles), Δ*arcE*-Pemp (white diamonds), Δ*arcE*-ParcE (red diamonds). (***C***) Effects of *arcB* complementation on growth without pyrimidine was evaluated in ASW-AA-m1-S^0-Pyr. Symbols: KPD1-Pemp (white circles), Δ*arcB*-Pemp (white triangles), Δ*arcB*-ParcB (red triangles). (***D***) The growth of each strain was evaluated in ASW-AA-m1-S^0-Pyr-Ura. Symbols: KPD1-Pemp (white circles), Δ*arcB*-Pemp (white triangles), Δ*arcB*-ParcB (red triangles). Error bars indicate the SD values of three independent culture experiments.

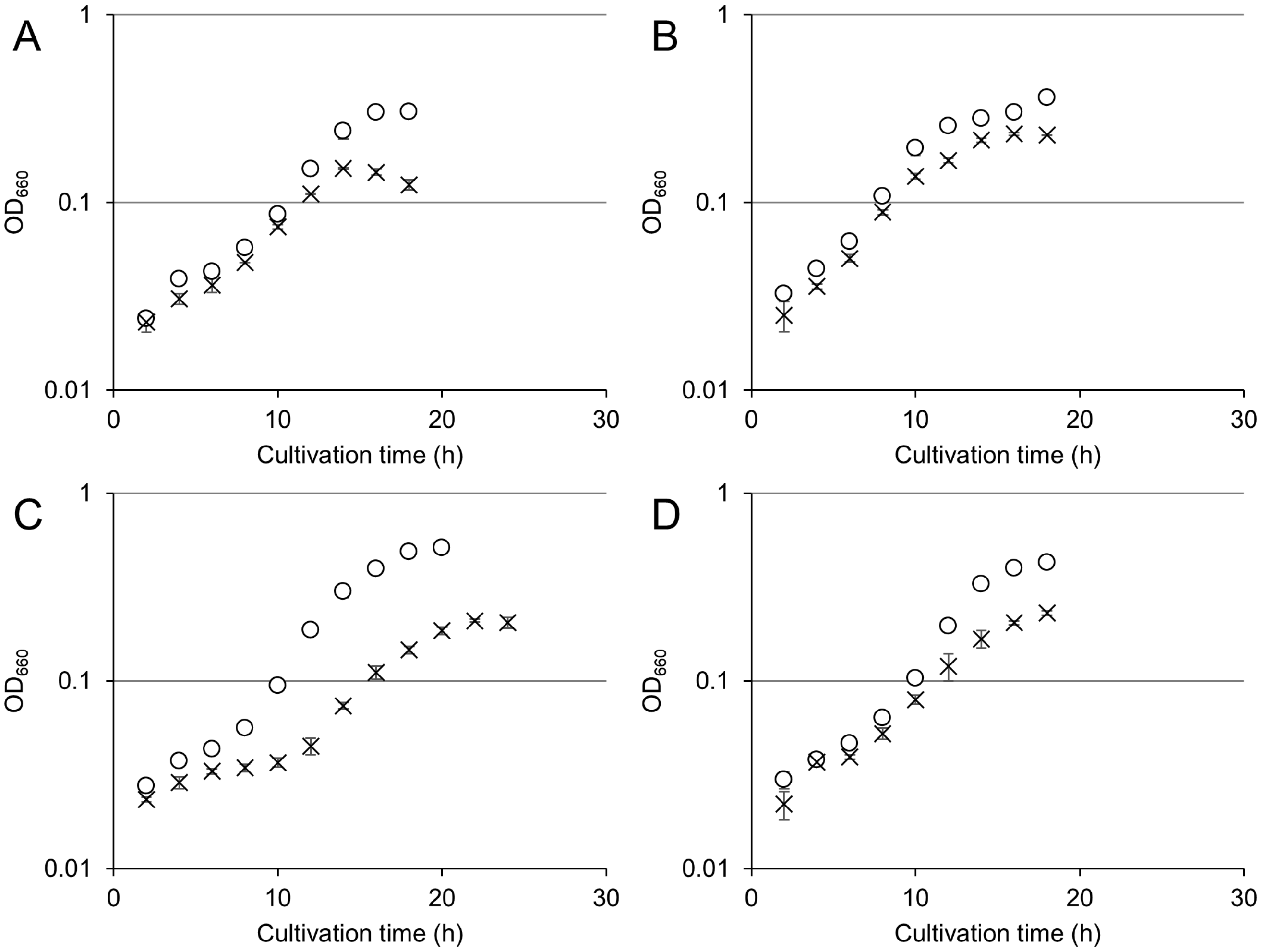

Supplementary Figure 4. Growth characteristics of *T. kodakarensis* strains under hydrogenase-dependent conditions. The growth of each strain was evaluated in ASW-YT-m1-Pyr or ASW-T-m1-Pyr media. ***A***, KPD1-Pemp; ***B***, KPD1-Pcps; ***C***, Δ*arcE*-Pemp; ***D***, Δ*arcE*-Pcps. Symbols: ASW-YT-m1-Pyr (circles), ASW-T-m1-Pyr (crosses). Error bars indicate the SD values of three independent culture experiments.
